# Incidence of brain metastasis according to patient race and primary cancer origin: a systematic review

**DOI:** 10.1007/s11060-024-04748-6

**Published:** 2024-06-19

**Authors:** David Gomez, Jeffrey J. Feng, Stephanie Cheok, Ishan Shah, Holly Dicharry, David J. Cote, Robert G. Briggs, Gage A. Guerra, Racheal Peterson, Bodour Salhia, Josh Neman, Frank Attenello, Frances Chow, Erion K. Musabelliu, Gabriel Zada

**Affiliations:** 1 Department of Neurosurgery Surgery, Keck School of Medicine, University of Southern California, Los Angeles, CA 90033 USA; 2 Western Michigan University Homer Stryker M.D. School of Medicine, Kalamazoo, MI USA; 3 Department of Neurological Surgery, Medical College of Wisconsin, Milwaukee, WI USA; 4 LSU Health Shreveport School of Medicine, Louisiana State University, Shreveport, LA 71103 USA; 5 Norris Comprehensive Cancer Center, University of Southern California, Los Angeles, CA 90033 USA; 6 Department of Neurology, Norris Comprehensive Cancer Center, Keck School of Medicine, University of Southern California, Los Angeles, CA USA; 7 Krembil Research Institute, University Health Network, and University of Toronto, Toronto, ON Canada

**Keywords:** Brain, Metastasis, Race, Disparities, Review, Incidence

## Abstract

**Purpose:**

A systematic review was conducted to investigate differences in incidence and primary origin of synchronous brain metastasis (sBM) in varying racial groups with different primary cancers.

**Methods:**

Adhering to PRISMA 2020 guidelines a search was conducted using PubMed and Ovid databases for publications from January 2000 to January 2023, with search terms including combinations of “brain metastasis,” “race,” “ethnicity,” and “incidence.” Three independent reviewers screened for inclusion criteria encompassing studies clearly reporting primary cancer sites, patient demographics including race, and synchronous BM (sBM) incidence.

**Results:**

Of 806 articles, 10 studies comprised of mainly adult patients from the United States met final inclusion for data analysis. Higher sBM incidence proportions were observed in American Indian/Alaska native patients for primary breast (*p* < 0.001), colorectal (*p* = 0.015), and esophageal cancers (*p* = 0.024) as well as in Asian or Pacific islanders for primary stomach (*p* < 0.001), thyroid (*p* = 0.006), and lung/bronchus cancers (*p* < 0.001) yet higher proportions in White patients for malignant melanoma (*p* < 0.001). Compared to White patients, Black patients had higher sBM incidence likelihood in breast cancer (OR = 1.27, *p* = 0.01) but lower likelihood in renal (OR = 0.46, *p* < 0.001) and esophageal cancers (OR = 0.31, *p* = 0.005). American Indian/Alaska native patients had a higher sBM likelihood (OR = 3.78, *p* = 0.004) relative to White patients in esophageal cancer.

**Conclusions:**

These findings reveal several comparative racial differences in sBM incidence arising from different primary cancer origins, underscoring a need for further research to explain these variations. Identifying the factors contributing to these disparities holds the potential to promote greater equity in oncological care according to cancer type.

**Supplementary Information:**

The online version contains supplementary material available at 10.1007/s11060-024-04748-6.

## Introduction

Brain metastasis (BM) remains a significant clinical challenge as the leading intracranial tumor in adults, with more than 100,000 new cases diagnosed annually [1, 2]. The National Cancer Institute’s Surveillance, Epidemiology, and End Results (SEER) database reports that of 1,872,057 cancer diagnoses between 2015 and 2019, 35,986 cases of synchronous BM (sBM) defined as BM identified at diagnosis of primary cancer were identified [3]. This singular statistic provides a crucial snapshot of BM at diagnosis, but likely underrepresents the total burden of metastatic disease, as BM is a progressive phenomenon developing at various timepoints throughout a patient’s disease course [3]. Measuring BM incidence remains a significant challenge due lack of standardized reporting in data registries with sBM often being the only included incidence metric. While sBM more accurately reflects cross-sectional prevenance, it remains the primary reported incidence measure in large data registries, providing crucial insights into the distribution of BM especially given that median survival for BM patients varies widely from 3 to 15 months and beyond, depending on primary cancer site [2–4].

Recent work reveals important disparities in BM incidence, particularly for the most common primary cancer origins known to cause BM, such as lung and breast cancer, with lifetime BM incidence rates occurring in 39–50% of lung cancer cases and 17–30% of breast cancer cases with variability across racial groups [5, 6]. Understanding the extent and distribution of these rates across racial groups is critical as other literature demonstrates disparity in access and outcomes based on race, such as Black patients experiencing longer hospitalization, higher in-hospital mortality rates, and non-routine discharge when compared to other races [7, 8].

Despite this evidence, trends in BM incidence for different racial groups diagnosed with different forms of cancer have yet to be explored in detail. Current literature, while extensive, lacks comprehensive reporting on BM for these groups, underscoring a pressing need for detailed exploration into BM incidence relating to race, based on primary cancer origin.

## Methods

### Search strategy

Following the Preferred Reporting for Systematic Reviews and Meta-Analysis (PRISMA) 2020 guidelines, a comprehensive search of the literature was performed. PubMed and Ovid databases were queried for articles published between January 2000 and January 2023. Identification of articles was conducted using PRISMA search recommendations and a thorough review of reference lists to capture relevant studies not present in initial database queries. Boolean search terms included were: (“brain metastasis” OR “brain neoplasms” OR “secondary brain tumor”) AND (“ethnicity” OR “race” OR “geographic region”) AND (“incidence”) AND (“impact” OR “role” OR “effect”). This search strategy yielded 806 total articles, with 323 from PubMed and 483 from Ovid.

### Study selection

Selection of articles involved three independent reviewers using a shared electronic spreadsheet to track and assess article inclusion. Articles were sorted by reported primary cancer type and organized by reviewer-assigned categories including *Lung*, *Breast*, *Genitourinary*, *Prostate*, *Skin*, and *Thyroid*. Duplicative studies identified through both database queries were noted and discarded throughout the study selection and review process. References of studies were cross-checked to ensure no additionally relevant studies were omitted.

### Inclusion and exclusion

Inclusion criteria consisted of: (1) Studies primarily analyzing brain metastasis with clearly documented identification pertaining to primary cancer sites; (2) Studies providing detailed patient information, including patient race and BM incidence; and (3) Full-text availability in the English language.

Exclusion criteria included: (1) Studies omitting clear specification of primary cancer site within a race group; (2) Studies reporting BM as a prospective statistic, without inclusion of present BM incidence over a given period; (3) non-peer reviewed articles, abstracts, editorials, or letters not available in the English language.

## Data extraction and analysis

**Fig. 1:**
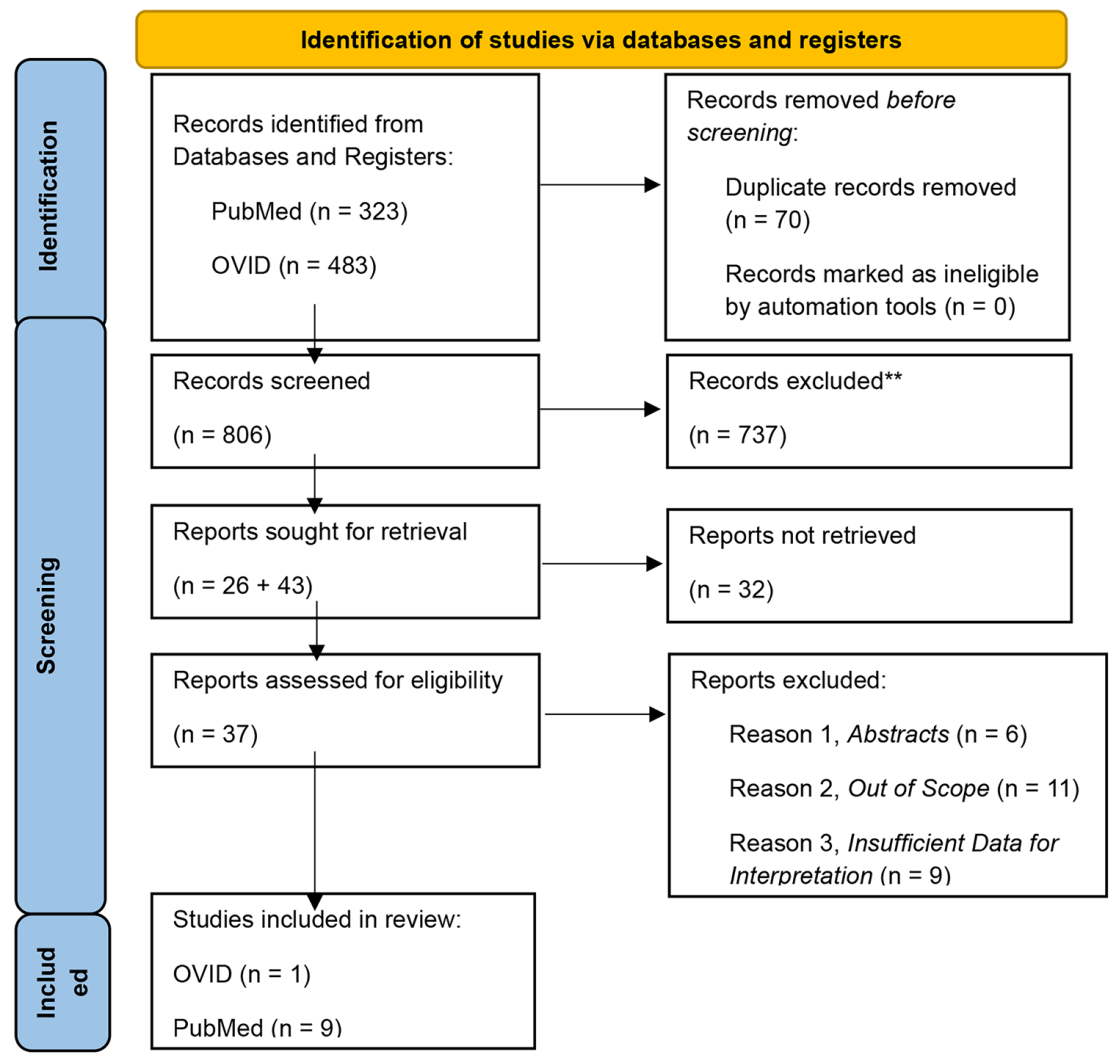
PRISMA flowchart depicting article retrieval and exclusion From: Page MJ, McKenzie JE, Bossuyt PM, Boutron I, Hoffmann TC, Mulrow CD, et al. The PRISMA 2020 statement: an updated guideline for reporting systematic reviews. BMJ 2021;372:n71. 10.1136/bmj.n71

From the selected articles, key variables including author lists, years of publication, central aims/findings, study design, and demographic information on BM incidence and statistical analysis or reports of significance were collected when available. Attention was given to the methodology of reported BM by race, regardless of statistical significance, for each primary cancer site. Following variable collection, a PRISMA flow chart depicting retrieval and selection was generated using the PRISMA flow diagram template (Fig. 1) [9].

**Fig. 2:**
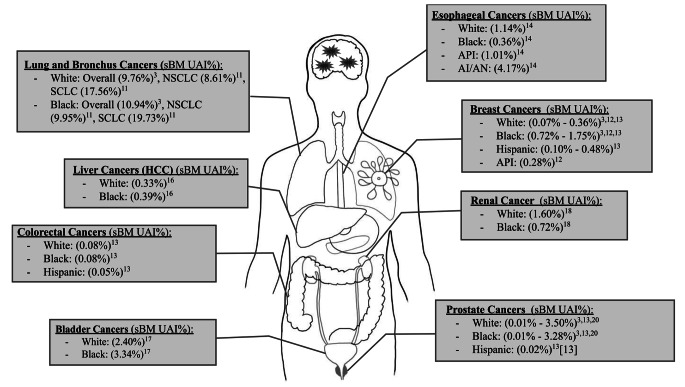
Unadjusted incidence percentages of brain metastases (sBM) by race and primary cancer type. Unadjusted incidence percentages (UAI%) of synchronous brain metastases (sBM) by race across various primary cancer sites. Data derived from multiple studies, as described in the footnotes below the figure. Percentages calculated as ratio of sBM cases with primary cancer by total primary cancer cases in each race group, based on data in Table 3. Illustration made using IPAD OS Freeform App (Apple), Microsoft Paint, and PowerPoint. Figure is original and no license is needed Data derived from references [3, 11–14, 16–18, 20]; sBM: Synchronous Brain Metastasis, UAI%: Unadjusted Incidence Percentage; API: Asian Pacific Islander; AI/AN: American Indian/Alaska Native; NSCLC: Non-Small Cell Lung Cancer; SCLC: Small Cell Lung Cancer; HCC: Hepatocellular Carcinoma

A summary of selected studies, including primary author, years of publication and databases utilized alongside key findings pertaining to BM incidence are outlined in Table 1. Risk of bias was assessed using the Newcastle Ottawa Scale (NOS), assigning points based on three broad domains (Table 2) [10]. Due to inconsistent or insufficient reporting of patient counts by age, race, BM or primary cancer status, age-adjusted incidence rates (AAIR) could not be calculated. Instead, unadjusted incidence was calculated for each race group and primary cancer population. Total counts of patients by BM status within racial category and primary cancer group are outlined in Table 3, alongside unadjusted incidence rates per 100,000. Unadjusted incidence percentages were then collected and represented visually in Fig. 2. Incomplete or unreported data was excluded from analysis.

**Table 1:** Summary of studies included in the systematic review: Summary of key findings for final selected studies. Variables include, Author and year of publication, database utilized by the study alongside the years from which data was analyzed, total number of patients included in the study, racial groups included, primary cancer types included, and key findings relating to brain metastasis incidence. The terms “sBM” and “BM” are reported per the original authors. Studies clearly denoting “sBM” as opposed to “BM” are noted with symbol (*)

Author, Publication Year	Database Studied (Years Analyzed)	Total Number of Patients	Racial Groups Included	Primary Cancer Types Evaluated	Key Findings
Parker, et al., 2023*	SEER (2015–2019)	1,872,057	White (Non-Hispanic), Black (Non-Hispanic), Hispanic, Asian/Pacific Islander, American Indian/Alaska Native	Lung and Bronchus, Melanoma, Breast, Kidney, and Renal Pelvis, Colorectal, Esophagus, Pancreas, Prostate, Stomach, Liver, Urinary Bladder, Ovary, Thyroid, Cervix	API patients: highest sBM% for primary stomach (*p* < 0.001), thyroid, (*p* < 0.001), and lung and bronchus cancers (*p* < 0.001). AI/AN patients: highest sBM% for primary breast (*p* < 0.001), colorectal (*p* = 0.015), esophageal (*p* = 0.024), kidney and renal pelvis cancers (*p* < = 0.001) Black patients: highest sBM% for prostate cancers (*p* = 0.028). White patients: highest sBM% for primary malignant melanoma (*p* < 0.001).
Akinyemiju, et al., 2018	SEER (2010–2013)	520,147	White (Non-Hispanic), Black (Non-Hispanic), Hispanic	Breast, Colorectal, Prostate	Higher odds of BM incidence in primary breast for Black patients compared to White patients (OR = 2.26, CI: 1.57–3.25). No significant association between race and BM incidence in primary colorectal cancer for Black (OR = 1.12) and Hispanic patients (OR = 0.63) compared to White patients.
Goncalves, et al., 2016	SEER (1973–2011)	34,681	White, Black	Lung Cancer Subtypes: NSCLC, SCLC	No significant differences in BM incidence between Black and White patients for NSCLC (OR: 1.03, *p* = 0.5) and SCLC (OR: 1.12, *p* = 0.27)
Martin, et al., 2017	SEER (2010–2013)	246,343	White, Black, Hispanic, Asian, Other	Breast Cancer Subtypes: HR+/HER2−, HR+/HER2+, HR−/HER2+, Triple-negative	Higher overall odds of BM incidence in primary breast cancer for Black patients compared to White (OR = 1.27, *p* = 0.01), alongside worse overall survival (HR = 1.34, *p* = 0.01)
Cheng, et al., 2021	SEER (2010–2018)	34,107	White, Black, Asian or Pacific Islander, American Indian/Alaska Native	Esophageal	Lower odds of BM incidence for Black patients (OR = 0.31, *p* = 0.005) and higher odds for AI/AN patients (OR = 3.78, *p* = 0.004) compared to White patients.
McGovern, et al., 2021	Single Institutional Cohort	264	White (Non-Hispanic), Hispanic, Black (non-Hispanic), Asian, unknown.	Colorectal	No significance for higher BM incidence in API patients for primary colorectal cancer, despite higher cohort rates compared to historical controls and autopsy studies.
Lin, et al., 2020	SEER (2010–2016)	43,310	White, Black, Other, Unknown	Hepatocellular Carcinoma	No significance for BM incidence by race in primary hepatocellular carcinoma (OR = 1.022, *p* = 0.927).
Rosiello, et al., 2020	NIS (2008–2015)	5,767	White, Black	Bladder	No significant association found between BM incidence and race, when comparing Black patients and White patients (OR: 1.23, *p* = 0.4) in primary bladder cancer.
Sun, et al., 2019	SEER (2010–2013), NCDB (2010–2012)	114,405	White, Black, Other, Unknown	Renal Cell Carcinoma	Lower odds of BM incidence in Black patients compared to White patients in primary renal cell cancer (OR = 0.46, *p* < 0.001).
Stolzenbach, et al., 2020	NIS (2008–2015)	6,963	White, Black	Prostate	No reported differences BM incidence by race for metastatic prostate cancer using NCDB data.

SEER: Surveillance, Epidemiology, and End Results; NIS: Nationwide Inpatient Sample; NCDB: National Cancer Database; NSCLC: Non-Small Cell Lung Cancer; SCLC: Small Cell Lung Cancer; HR+: Hormone Receptor-Positive; HER2: Human Epidermal Growth Factor Receptor 2

**Table 2:** Risk of bias assessment for selected studies: Risk of Bias using the Newcastle-Ottawa Scale (NOS) for nonrandomized studies. Scores based on three main criteria: Selection (representativeness of cohort, selection of non-exposed cohort, exposure ascertainment, outcome of not interest present at start), Comparability (confounding control measures), and Outcome (assessment, follow-up duration, and adequacy)

Study	Selection (max 4)	Comparability (max 2)	Outcome (max 3)	NOS Score (max 9)
Akinyemiju, et al., 2018	***	**	**	7
Cheng et al., 2021	***	**	**	7
Goncalves et al., 2016	****	**	**	8
Lin et al., 2020	****	**	**	8
Martin et al., 2017	****	**	***	9
McGovern et al., 2021	***	**	**	7
Parker et al., 2023	****	**	***	9
Rosiello et al., 2020	****	**	***	9
Stolzenbach et al., 2020	***	**	**	7
Sun et al., 2019	***	**	**	7

Each (*) indicating one point toward each criterion within categories.

**Table 3:** Distribution of synchronous brain metastases (sBM) by race and primary cancer type across various studies. Summary of patients by primary cancer cases overall and by sBM for each race group. Data derived when not directly reported. Unadjusted Incidence percentages (UAI %), calculated where available, as a ratio and percent product for sBM cases over total cases in each primary site and race group. Studies not reporting necessary metrics are denoted with superscript (a). Studies clearly denoting “sBM” as opposed to “BM” are noted with superscript (b), however all UAI% are from databases that report sBM. Missing or incalculable data denoted by symbol (-)

Author, Year	Primary Cancer Type	Race-Specific Primary Cancer Cases (n)	Race Group: *BM incidence per 100,000*	WhitesBM Cases (UAI %)	Black sBM Cases (UAI %)	*API* sBM Cases (UAI %)	*AI/AN* sBM Cases (UAI %)	Hispanic sBM Cases (UAI %)
Parker, 2023 ^**(a) (b)**^	Lung and Bronchus	-	-	21,357	3269	1852	181	-
	Melanoma	-	-	1474	12	11	2	-
	Breast	-	-	1021	201	77	16	-
	Stomach	-	-	170	16	13	6	-
	Thyroid	-	-	56	12	0	13	-
	Prostate	-	-	170	44	2	7	-
	Bladder	-	-	113	4	2	2	-
	Renal Pelvis and Kidney	-	-	936	93	15	42	-
	Liver	-	-	113	20	2	7	-
	Pancreatic	-	-	199	32	3	18	-
	Esophageal	-	-	340	24	8	11	-
	Colorectal	-	-	425	64	6	24	-
Goncalves, 2016	Lung (NSCLC)	White (23,773), Black (6673)	White: *8,614*; Black: *9,951*	2048 (8.61%)	664 (9.95%)	-	-	-
	Lung (SCLC)	White (3485), Black (750)	White: *17,556*; Black: *19,733*	612 (17.56%)	148 (19.73%)	-	-	-
Akinyemiju, 2018	Breast	White (159,933), Black (2,405), Hispanic (23,181)	White: *71*; Black: *1,746*; Hispanic: *99*	115 (0.07%)	42 (1.75%)	-	-	23 (0.10%)
	Colorectal	White (93,056), Black (16,576), Hispanic (14,174)	White: *82*; Black: *84*; Hispanic: *49*	77 (0.08%)	14 (0.08%)	-	-	7 (0.05%)
	Prostate	White (139,777), Black (30,914), Hispanic (18,503)	White: 7; Black: 9; Hispanic: 16	11 (0.008%)	3 (0.01%)	-	-	3 (0.02%)
Martin, 2017	Breast	White (165,808), Black (25,914), API (19,043), Hispanic (25,359)	White: *364*; Black: *721*; API: *278*; Hispanic: *477*	604 (0.36%)	187 (0.72%)	53 (0.28%)	-	121 (0.48%)
Cheng, 2021	Esophageal	White (15,665), Black (1675), API (887), AIAN (120)	White: *1,136*; Black: *358*; API: *1,014;* AI/AN: *4,166*	178 (1.14%)	6 (0.36%)	9 (1.01%)	5 (4.17%)	-
McGovern, 2021^(**a**)^	Colorectal	White (28), Black (26), Hispanic (26), API (76)	-	-	-	5	-	-
Lin, 2020	Liver (HCC)	White (29,965), Black (6095)	White: *330*; Black: *393*	99 (0.33%)	24 (0.39%)	-	-	-
Rosiello, 2020	Bladder	White (5169), Black (598)	White: *2,398*; Black: *3,344*	124 (2.40%)	20 (3.30%)	-	-	-
Sun, 2019	Renal Pelvis and Kidney	White (29,492), Black (4310), Other/Unknown (2,577)	White: *1,600*; Black: *719*	472 (1.60%)	31 (0.72%)	-	-	-
Stolzenbach, 2020	Prostate	White (3,881), Black (1,494)	White: *3,503*; Black: *3,279*	136 (3.50%)	49 (3.28%)	-	-	-

(a): Unadjusted incidence % unable to be calculated; (b): Denoted “sBM” specifically; (-): Value not reported or unable to be obtained

UAI %: Unadjusted Incidence Percentage API: Asian Pacific Islander, AIAN: American Indian/Alaska Native, NSCLC: Non-Small Cell Lung Cancer, SCLC: Small Cell Lung Cancer, HCC: Hepatocellular Carcinoma,

## Results

### Overview of study screening and selection

Our systematic review yielded a total of 806 articles identified through our search strategy and screening. Following eligibility-based inclusion and exclusion criteria assessment, 75 articles were deemed potentially relevant (Fig. 1). Further exclusion, due to insufficient data on BM incidence by race specifically, was conducted on full text and abstract review. Overall, 10 articles, comprised of United States patient databases, met full criteria for inclusion (Table 1).

### Characteristics of included studies

The final 10 articles predominantly encompassed observational and retrospective study designs, with 7 studies utilizing the SEER database, which reports sBM as the main incidence metric (Table 1). However, the terms “BM” and “sBM” were often used interchangeably posing a challenge for analysis. Ultimately, we utilized the terms “BM” and “sBM” as per original author accounts in our results but denote the more accurate designation of sBM in the included tables and figures unless specifically indicated. Selected studies spanned publication years 2000 to 2023, with patient data ranging from 1973 to 2019 (Table 1). Risk of bias scores ranged from 7 to 9, indicating low to moderate risk of bias across all categories (Table 2).

## Detailed results from included studies by primary cancer site/origin

### Lung cancer

In a 2023 study by Parker et al., a total of 1,872,057 patients with malignant cancer from 2015 to 2019 available in the SEER database were analyzed to evaluate trends in sBM incidence. When reporting sBM by race, for each primary cancer site, sBM percentages (sBM %) represented relative frequency in each race group rather than specific rates in the total population limiting derivation of unadjusted sBM incidence rates (Table 3) [3]. Lung and bronchus cancers comprised the largest proportion of sBM patients (*n* = 27,585) with an incidence of 7.1 per 100,000 persons [3]. For this group, sBM% was highest in Asian or Pacific Islander (API) patients at 84.3%, followed by Black (81.2%), White (75.3%), and American Indian/Alaska Native patients (AI/AN) (66.8%) (*p* < 0.001) [3].

Goncalves et al. assessed 30,446 non-small cell lung cancer (NSCLC) and 4,235 small cell lung cancer (SCLC) cases from the SEER database. For NSCLC, higher BM incidence was observed for Black patients (10%) compared to White patients (9%) (OR:1.03, 95% CI: 0.94–1.13, *p* = 0.5) but lower BM incidence in SCLC (OR: 1.12, 95% CI: 0.92–1.37, *p* = 0.27) (Fig. 2) [11].

### Breast cancer

Parker et al. reported 1,369 primary breast cancer patients with sBM at an incidence of 0.24 to 0.30 per 100,000 patients [3]. AI/AN patients had the highest sBM% at 5.9% compared to Black (5.0%), White (3.6%), and API patients (3.5%) (*p* < 0.001) [3].

Using SEER, Martin et al. studied 968 BM patients with primary breast cancer, of which 848 were included for survival analysis. Unadjusted incidence percentages were highest in Black patients (0.72%), followed by White (0.36%), Hispanic (0.48%), and API (0.28%) patients (Fig. 2). Comparison of Black patients revealed significantly greater odds of BM incidence (OR = 1.27, 95% CI: 1.06–1.53, *p* = 0.01) compared to White patients and worse overall survival (HR = 1.34, 95% CI: 1.06–1.69, *p* = 0.01) when adjusting for socioeconomic factors and extent of disease [12].

Using SEER, Akinyemiju et al. included 180 BM patients with primary breast cancer. Unadjusted incidence percentage was highest for Black patients (1.75%) followed by Hispanic (0.10%) and White (0.07%) patients (Fig. 2.). Black patients had a higher odds of BM incidence compared to White patients (OR = 2.26, 95% CI: 1.57–3.25, α = 0.05, *p* = not reported) [13].

### Gastrointestinal and hepatic cancers

In Parker et al. significant differences in sBM% were noted for AI/AN patients in primary colorectal cancer (2.2%) compared to White (1.5%), Black (1.6%), and API patients (1.1%) (*p* = 0.015) [3]. Similarly, in primary esophageal cancer patients sBM% was highest in AI/AN patients at 3.0% compared to all other race groups (0.5%, *p* = 0.024) [3]. API patients, however had higher sBM% (0.6%) in primary stomach cancer (*p* < 0.001) [3].

Akinyemiju et al. included 98 BM patients with primary colorectal cancer. Unadjusted incidence percentages were 0.08% for both White and Black patients but 0.05% for Hispanic patients (Fig. 2.). Race was not significantly associated with BM incidence for both Black (OR = 1.12; CI: 0.63–1.99, *p* = not reported) and Hispanic patients (OR = 0.63; CI: 0.29–1.37, *p* = not reported) when compared to White patients on multivariable regression [13].

Using SEER, Cheng et al. reports 181 sBM patients with primary esophageal cancer. Unadjusted incidence percentages were 1.14% for White patients followed by 0.36% for Black patients, 1.01% for API patients, and 4.17% for AI/AN patients (Fig. 2.). Compared to White patients univariable logistic regression analysis revealed lower odds of BM incidence for Black patients (OR = 0.31, 95% CI: 0.12–0.65; *p* = 0.005) but higher BM odds for AI/AN patients [14].

McGovern et al. analyzed a single institution retrospective cohort of 264 patients with primary colorectal cancer from which 5 API patients (7%) developed BM, which was higher than historical controls (0.6 − 3.2%) and autopsy studies of BM (0.9-2.7%)– however they report no significance [15].

Lin et al. included 121 primary hepatocellular carcinoma patients with BM from the SEER database. Calculated incidence percentage was 0.33% for White patients and 0.39% for Black patients (Fig. 2.). Multivariable logistic regression revealed no significant associations for BM incidence by race (OR = 1.022, 95% CI: 0.643–1.624, *p* = 0.927) [16].

### Genitourinary cancers

Parker et al. included 1,100 (3.1%) patients with primary kidney and renal pelvis cancers and 119 (0.3%) primary bladder cancer patients within their sBM cohort. AI/AN patients showed significantly higher sBM% for kidney and renal pelvis cancers at 5.5%, followed by White patients (3.3%), Black patients (2.3%), and API patients (1.9%) (*p* = 0.001) [3]. Similarly higher sBM% rates were seen for primary bladder cancer in AI/AN patients but were not significant (*p* = 0.129) [3].

Rosiello et al. identified metastatic bladder cancer patients from the National Inpatient Sample Database (NISD). Calculated incidence of BM was 2.4% for White patients and 3.3% for Black patients (Fig. 2.). Compared to White patients, Black patients had no association for BM incidence despite positive associations present in other metastatic sites (OR: 1.23, 95% CI: 0.74–2.05, *p* = 0.4) [17].

Sun et al. reported both SEER (2010–2013) and National Cancer Database (NCDB) data (2010–2012) for primary renal cell cancer (RCC). However, only SEER patients were included for full statistical analysis [18]. Unadjusted incidence percentages for SEER patients were 1.6% for White patients and 0.7% for Black patients (Fig. 2.) [18]. On bivariate analysis, Black patients had a lower odds of BM incidence compared to White patients (OR = 0.46, 95% CI: 0.32–0.67, *p* < 0.001) [18]. Bootstrap-corrected multivariable logistic regression revealed higher BM odds in White patients, compared to Black patients (OR = 1.87, 95% CI: 1.29–2.71, *p* = 0.001) [18]. The authors also included “Other” and “Unknown” race categories, which SEER defines as “*Other*” being a category for cases not captured by primary classification (mixed race, or not “White”, “Black”, “API”, or “AI/AN”) while “*Unknown*” is used when data is not present [19]. “Other” race was associated increased odds of BM, on multivariable logistic regression, when compared to Black race (OR = 1.99, 95% CI: 1.25–3.17, *p* = 0.004) [18].

### Prostate cancer

Parker et al. reported 237 prostate cancer patients with sBM, with SBM% highest in Black patients (1.1%) (*p* = 0.028) [3]. In contrast, Akinyemiju et al. reported no significance in BM incidence when comparing Black and Hispanic patients to White patients (OR = 1.67, 95% CI: 0.46–6.06, OR = 2.19, 95%CI: 0.61–7.86, *p*-values not reported) [13]. Unadjusted incidence percentage was highest for Black patients (0.08%) (Fig. 2.).

Stolzenbach et al. examined 6,963 patients with metastatic prostate cancer using the NCDB database (2008–2015) from which the unadjusted incidence percentage was higher in White patients (3.50%) compared to Black patients (Fig. 2.). However, no significance for BM incidence by race was reported [20].

### Skin cancer

Parker et al. reported significantly higher sBM% for White patients with primary malignant melanoma (5.2%) followed by AI/AN (0.7%), API (0.7%), and Black patients (0.3%) (*p* < 0.001) [3].

### Thyroid

For primary thyroid cancer, Parker et al. reported significantly higher sBM% in API patients followed by Black (0.3%), and White (0.2%) patients (*p* = 0.006) [3].

## Discussion

Metastatic lesions to the brain remain a common problem for the aging, adult population in the United States [1, 2]. Measuring incidence of BM from different primary cancers can help clinicians and scientists better understand the scope of the problems encountered in treating these patients and explore any potential racial differences contributing to BM predilection seen in certain primary cancers. This, in turn, may guide research in management, screening, and targeted cancer therapy. Moreover, quantifying differences in disease burden by race elucidates present disparities in healthcare access, diagnosis and treatment for cancer patients with BM. To that end, this systematic review summarizes the BM incidence according to various primary cancers arising in different racial groups.

Our results highlight important differences in the frequency and type of cancers occurring in different races and their association with the variable development of BM. The studies in this systematic review collectively reveal (1) statistically higher likelihood of BM incidence for Black patients compared to White patients in primary breast cancer [11, 12] (2) significantly lower likelihood of BM incidence in primary esophageal cancer for Black patients compared to White patients but higher likelihood of BM incidence for AI/AN patients compared to White Patients [13] (3) lower likelihood of BM incidence in primary renal cell cancer for Black patients compared to White patients [18] (5) and when comparing sBM% between White, Black, AI/AN and API patients, as distributions across primary cancer sites, highest sBM% was seen for API patients with primary stomach, thyroid, and lung and bronchus cancers vs. AI/AN patients with primary breast, colorectal, esophageal, kidney and renal pelvis cancers, yet higher rates in Black patients with primary prostate cancer and White patients with primary malignant melanoma [3]. These findings underscore the need for accurate prospective demographic data collection on race and ethnicity to better examine BM incidence by primary cancer type.

Recent literature substantiates that racial and ethnic minorities face disproportionate burdens of cancer-related morbidity and mortality for both primary brain cancer and metastatic disease, the key findings of this review elucidate composite trends in metastatic cancer to the brain, likely driven by the complex interplay of biological, genetic, cultural, socioeconomic, and systemic factors, among others [21, 22]. Our results build on previous work by revealing increased BM incidence in AI/AN, API, and Black patients for certain cancers. This is especially relevant given other studies showing lower cancer-directed therapy in AI/AN patients with metastatic cancer alongside higher complication rates for Black patients with BM [23, 24].

### Limitations

This systematic review is limited by the retrospective nature of the data drawn from each study and the use of shared cancer databases between studies, resulting in some overlap in outcomes reporting. Retrospective analysis of these studies also limits our ability to capture dynamic information related to brain cancer progression and the development of new BM. Advanced meta-analytic methods would have allowed data pooling across studies and increased statistical power to assess these trends but were not possible, in large part due to the heterogeneity in reporting outcomes, racial grouping, and statistical methods across studies.

A significant limitation is potential misrepresentation of racial disparities due to confounding factors such as healthcare access. Poor access to healthcare, prevalent among the studied populations, may lead to later-stage disease presentations when BM is more likely to be detected, potentially obscuring the true incidence of BM. Additionally, racial categorization between studies lacks nuance and fails to reflect true genetic diversity limiting conclusions on the effect of the characteristics on BM. Future work, will benefit from prospective research designs with standardized arrays of demographic and biological variables to better measure influence and incidence of BM in different racial groups.

Finally, it is critical to understand the distinction between studies reporting sBM versus all BM with the former term capturing patients diagnosed with BM at the time of initial cancer diagnosis versus the latter capturing patients with BM at any point during their disease course. This variability in reporting was present across all studies and 7 of 10 studies in our final selection utilized the SEER registry which reports sBM as the primary incidence metric. However, this distinction as not made consistently clear which limited our ability to conduct more robust analysis. Importantly, these findings elucidate the crucial need for accurate reporting of synchronous versus asynchronous metastasis to truly classify the extent of BM due to race, poor access to care, or a combination of other differences.

Regardless of these limitations, this work compiles the currently known trends in BM incidence across race and primary cancer while highlighting crucial areas of future research to bridge our remanning gaps in knowledge in cancer care.

## Conclusions

This systematic review summarizes the recent literature regarding BM incidence stratified by race for various primary cancer sites. The data presented highlights significant differences in BM incidence for various racial groups across primary cancer origin reflecting the critical need for targeted epidemiological and clinical analysis to elucidate the underlying cause of these disparities. Understanding and mitigating the factors that contribute to these differences holds the potential to enhance screening protocols, modalities for treatment, and equity thereby advancing oncologic care for all.

### Electronic supplementary material

Below is the link to the electronic supplementary material.

Supplementary Material 1

## Data Availability

No datasets were generated or analysed during the current study, other than those reported in the tables and figures.
